# Integrated transcriptomic and metabolomic analyses reveal the regulatory network of alkaloid metabolism in blister blight-infected tea plants

**DOI:** 10.3389/fpls.2026.1885792

**Published:** 2026-08-05

**Authors:** Yanglongyu Chen, Ping Li, Yuqing Ma, Song Tang, Chenyu Mao, Jiaqi Liu, Xiaolu Zhou, Caibi Zhou

**Affiliations:** 1 Key Laboratory of National Forestry and Grassland Administration on Biodiversity Conservation in Southwest China; Yunan Academy of Biodiversity; Key Laboratory of Forest Disaster Warning and Control in Yunnan Province; Ancient Tea Tree Research Center; College of Forestry, Southwest Forestry University, Kunming, China; 2 State Key Laboratory of Discovery and Utilization of Functional Components in Traditional Chinese Medicine, School of Pharmaceutical Sciences, Guizhou Medical University, Guiyang, China; 3 Tea Research Institute, Yunnan Academy of Agricultural Sciences, Kunming, China

**Keywords:** blister blight disease, *Exobasidium vexans*, isoquinoline alkaloid biosynthesis, metabolome profiles, tea plant, transcriptome analysis

## Abstract

Blister blight disease, caused by the fungus *Exobasidium vexans*, severely threatens tea production and yield. This study investigates the molecular and metabolic responses of tea to blister blight infection, with a focus on alkaloid metabolism. Here, integrated transcriptomics and metabolomics were employed to investigate the differences between healthy leaves and infected leaves at three disease stages. In total, 79 alkaloid metabolites were identified, with 30 differential metabolites shared across all infection stages. Transcriptome sequencing revealed 846 differentially expressed genes, many of which were enriched in the isoquinoline alkaloid biosynthesis pathway. Key structural genes (e.g. *TAT*, *PPO*) and transcription factors (e.g., *WRKY*, *GRAS*) were significantly upregulated during infection, correlating with altered alkaloid profiles. Notably, dopamine, a key intermediate, was downregulated, suggesting a potential shift in metabolic flux toward downstream defense-related alkaloid synthesis. qRT-PCR validation confirmed the expression patterns of selected DEGs. Our findings provide the first comprehensive evidence that alkaloid metabolism, particularly the isoquinoline alkaloid pathway, are transcriptionally and metabolically reprogrammed during blister blight infection, suggesting a potential role in tea’s defense against this pathogen. These results offer valuable insights for breeding resistant tea cultivars.

## 1 Introduction

Tea plant (*Camellia sinensis* L.), is a perennial evergreen crop of considerable economic and cultural significance, providing the primary source of globally consumed non-alcoholic beverages. In addition to its agronomic importance, it harbors diverse specialized metabolites, particularly polyphenols, carbohydrates, amino acids, and alkaloids (e.g., caffeine), which are largely responsible for its distinctive flavor, aroma, and bioactivity (Li et al., 2021; Zhou et al., 2021). However, tea cultivation is challenged by biotic stressors, among which blister blight caused by the obligate biotrophic fungus *Exobasidium vexans* is particularly widespread across Asia’s major tea-growing regions, with severe impacts reported in India, China, Japan, Sri Lanka, and Indonesia (Sen et al., 2020; Mahadevan et al., 2024). This disease is prevalent across all tea-growing areas of China with the exception of the region north of the Yangtze River. The most severe outbreaks are observed in high-elevation tea plantations located in the southwestern and central-southern parts of the country, particularly in Guizhou, Sichuan, and Yunnan provinces (Yang et al., 2019). Blister blight predominantly infects young leaves and buds, inducing hypertrophic growth, chlorosis, and necrosis, which collectively reduce photosynthetic efficiency and cause yield reductions of 20–50% under severe epidemics (Pandey et al., 2021). While conventional fungicides like triazoles and strobilurins offer partial control, their overuse raises concerns about environmental contamination, pathogen resistance, and residue accumulation in tea products (Ajay and Baby, 2010). Consequently, unraveling the intrinsic defense mechanisms of tea plants, particularly those involving alkaloid metabolism, has emerged as a promising strategy for sustainable disease management and cultivar improvement.

The pathogen-induced reprogramming of host metabolism is typically coordinated by transcriptional networks that regulate defense-related pathways (Mansfeld et al., 2017). Alkaloids, a major class of nitrogenous secondary metabolites constituting about 20% of plant-specialized metabolites (Tang et al., 2023), are structurally classified into benzylisoquinoline, monoterpenoid indole, and tropane alkaloids (Zhou et al., 2024b). These compounds serve as constitutive or inducible chemical defenses, enhancing plant resilience to biotic stress. In tea, alkaloids are not only pivotal for quality traits but also possess antimicrobial and signaling properties (Chen et al., 2024; Dai et al., 2025). Caffeine, for instance, has been shown to compromise fungal plasma membrane integrity and suppress conidial germination (Delorme et al., 2025). However, the mechanistic intersection between alkaloid metabolic pathways and resistance to blister blight remains poorly understood.

The application of high-throughput sequencing and metabolomic profiling has markedly advanced thestudy of plant–pathogen dynamics (Li et al.,2025). Transcriptomic approaches (e.g., RNA-Seq) enables genome-wide identification of differentially expressed genes (DEGs) involved in defense signaling, while metabolomic techniques (e.g., LC-MS and GC-MS) provide a comprehensive insight into stress-induced metabolic shifts (Zhou et al., 2024a; Han et al., 2025). Integrative analysis of these datasets can unravel regulatory hubs where transcriptional changes directly influence metabolite accumulation, offering mechanistic insights into plant immunity (Kushalappa et al., 2016). Supporting evidence includes defense-related monoterpenoid indole alkaloid accumulation in elicited Uncaria tomentosa cultures (Correa-Higuera et al., 2019), *StWRKY8*-mediated regulation of benzylisoquinoline alkaloids in potato late blight resistance (Yogendra et al., 2016), and alkaloid gene expression shifts in *Lupinus angustifolius* upon anthracnose infection (Czepiel et al., 2021). Nevertheless, such multi-omics approaches have seldom been applied to fungal diseases regarding targeted alkaloid metabolism in tea. This gap hinders the development of biomarkers for breeding blight-resistant cultivars and limits understanding of how alkaloids synergize with other defense pathways during infection.

This study employs transcriptomics and widely targeted metabolomics to investigate the spatial and temporal reorganization of alkaloid metabolism in tea leaves following blister blight infection. We aim to identify blister blight-responsive DEGs and differentially accumulated metabolites (DAMs), with emphasis on alkaloid biosynthesis and regulatory genes; and construct co-expression networks linking transcriptional regulators to alkaloid metabolic pathways. We hypothesized that blister blight infection induced alkaloid response: an early phase marked by pathogen recognition and signal transduction, followed by sustained upregulation/downregulation of alkaloid synthesis genes to limit fungal proliferation. During the continuous infection process of blister blight, dynamic transcriptional and metabolic changes were captured. By correlating DEGs with DAMs, candidate genes and alkaloid metabolites that defined tea’s blight resistance phenotype were identified. Furthermore, functional validation was strengthened via qRT-PCR. This study presents a crucial molecular mechanism regarding tea plant in response to *E. vexans* infection and identifies critical alkaloid-related pathways involved in its resistance to pathogen infection, which are expected to significantly enhance the breeding program of resistant varieties. However, we acknowledge that the current metabolomic platform is widely targeted and may not capture all isoquinoline alkaloid derivatives, and that direct detection of pathway end products is limited; thus, the inferred pathway activation requires further targeted validation.

## 2 Materials and methods

### 2.1 Plant samples collection

**Figure 1:**
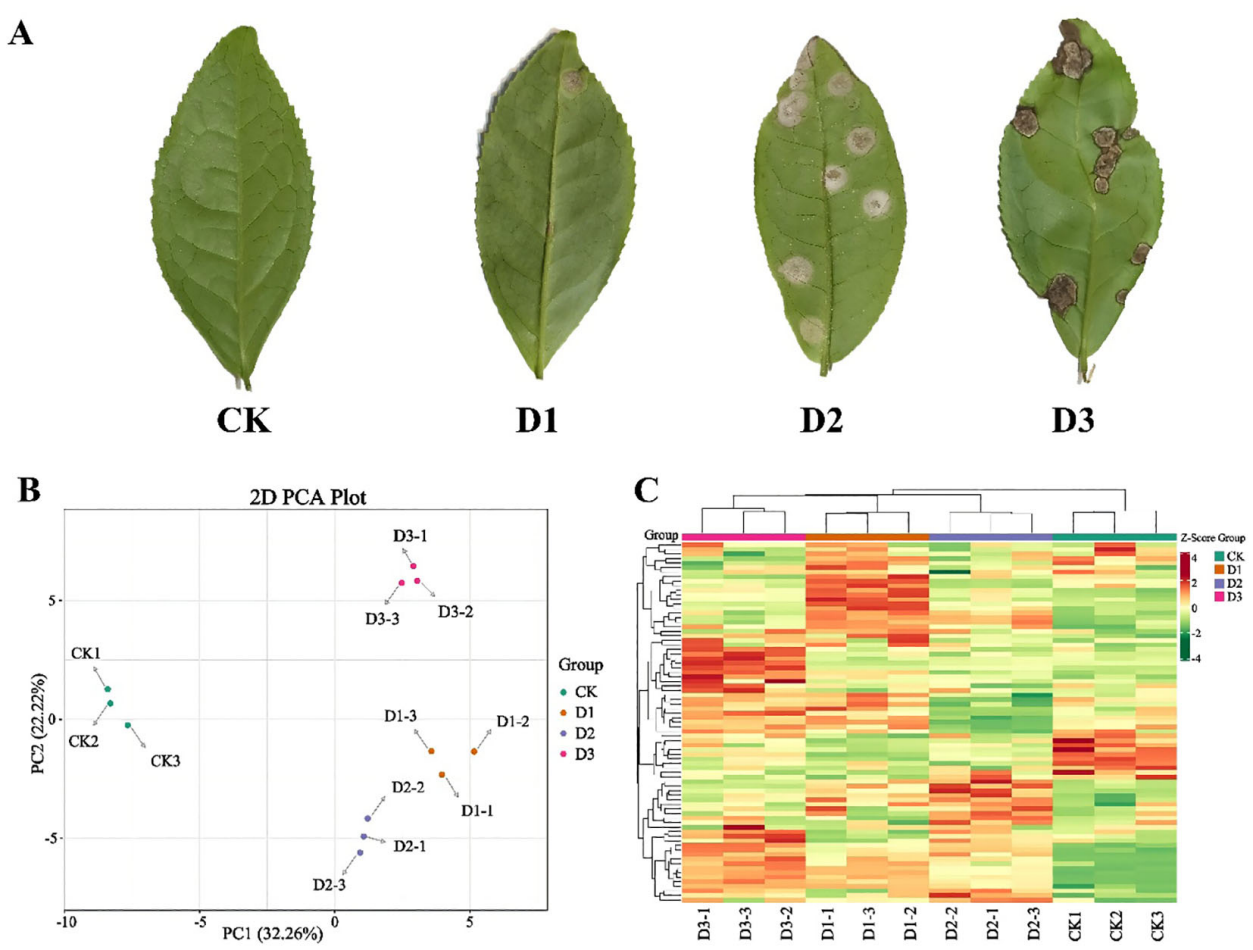
Blister blight symptoms and disease-induced alterations in tea alkaloid metabolites. **(A)** Healthy leaves (CK) and tea leave infected with blister blight at designated infection stages 1 (D1), 2 (D2), and 3 (D3). **(B)** Principal component analysis (PCA) of metabolites among the four groups. **(C)** Heatmap of metabolites in different degrees of infection.

Plant materials employed in this study were obtained from tea plantations located in Duyun City, Guizhou, China (1,576 m altitude; 106°49′E, 25°27′N). Tea plants were grown with a row spacing of 1.5 m and a cluster spacing of 25−33 cm under the conditions of an average temperature of 16°C and average precipitation of 1200 mL. For leaf collection, the third leaves of 15-year-old Fuding Dabaicha cultivar were randomly sampled from more than 30 tea bushes on a sunny morning in May. During this disease season, the prevalence of blister blight was favored by 20−22°C and high humidity (> 80%). The samples were divided into four groups based on macroscopic disease symptoms, following well-established criteria for blister blight progression (Pandey and Somssich, 2009; Sen et al., 2020): healthy leaves (CK) with no visible symptoms; infected leaves showing translucent spots (D1, 1–3 mm diameter, water-soaked appearance); infected leaves with mature blisters (D2, 5–10 mm diameter, pale green to yellowish protrusions); and infected leaves with necrotic spots (D3, central necrosis with brownish discoloration and chlorotic halo) (**Figure 1A**). All the leaves exhibited uniformity in size, without any signs of mechanical damage or other disease. The collected samples were promptly frozen using liquid nitrogen and subsequently stored at −80 °C for metabolome, transcriptome, and compound quantification analysis. Three biological replicates were conducted for each treatment.

### 2.2 Sample extraction

The samples were freeze-dried using Scientz-100F vacuum freeze-dryer and were then crushed for 1.5 min at 30 Hz using an MM 400 mixer mill (Retsch, Germany) equipped with a zirconia bead. Approximately 100 mg of lyophilized powder was dissolved in 1.2 mL of 70% methanol. The process of vortex extraction was repeated a total of six times, with each repetition lasting for 30 s, follow by placing overnight at 4 °C. The extracts were subsequently centrifuged at 12,000 × g for 10 min to collect the supernatant for filtration using a 0.22-μm Millipore membrane filter (SCAA-104; ANPEL, Shanghai, China, http://www.anpel.com.cn/) for an ultra-performance liquid chromatography electrospray ionization tandem mass spectrometry (UPLC-ESI−MS/MS) analysis.

### 2.3 Metabolites analysis

The sample extracts were performed on UPLC-ESI−MS/MS system (UPLC, SHIMADZU Nexera X2; MS, Applied Biosystems 4500 Q TRAP). The UPLC conditions were as follows: Agilent SB-C1 column (1.8 µm, 2.1 mm × 100 mm); mobile phase consisting of pure water with 0.1% formic acid (solvent A) and acetonitrile with 0.1% formic acid (solvent B); flow velocity, 0.35 mL/min; column oven temperature, 40 °C; injection volume, 4 μL. The determinations were conducted using a gradient program, which initially consisted of 95% A and 5% B; after 9 min, a linear gradient to 5% A and 95% B was applied, and was maintained for 1 min; following that, the composition was adjusted to 95% A and 5% B within 1.1 min and held constant for 2.9 min. AB4500 Q TRAP UPLC/MS/MS System were managed by Analyst 1.6.3 software (AB Sciex) in both positive and negative ion modes.

The samples of quality control (QC) were created by pooling together all individual samples to evaluate the reproducibility of the obtained data. Qualitative analysis of substances was conducted through the utilization of secondary spectral information using self-constructed database, MWDB (Metware Database). The signals from isotopes, as well as repeated signals of K^+^, Na^+^, NH^4+^, and fragment ions derived from larger molecules, were eliminated in the course of analysis. Quantitative analysis of metabolites was performed using the multiple reaction monitoring (MRM) mode of triple quadrupole (QQQ) mass spectrometry. For differential metabolite screening, significantly regulated metabolites in alkaloids among the four groups were screened out by variable importance of the projection (VIP) scores of ≥ 1 and absolute log2FC (fold change) ≥ 1. The stepwise screening procedure began with the initial identification of all detected metabolites from the MWDB database, followed by their classification into categories. For each comparison, DAMs were selected using VIP and fold change thresholds. For K-means clustering, the z-score-normalized relative abundances of all DAMs were grouped by the K-means algorithm based on Euclidean distance, with the optimal K value determined by the elbow method. And finally, a Venn diagram was used to visualize the overlapping DAMs among the three comparisons. The identified metabolites were annotated by using the KEGG compound database to establish the metabolic pathways that exhibited the significant correlation with alkaloids metabolites, which significance was determined by the *p*-values of hypergeometric test.

### 2.4 Transcriptome sequencing

Transcriptome analysis of tea leaves infected by bister blight disease was performed by Biomarker Technologies LTD (Beijing, China). Briefly, total RNA was extracted using the TRIzol^®^ reagent (Invitrogen, Carlsbad, USA) following the manufacturer’s protocols, after which the concentration and quality of RNA that met the requirements was estimated using Nanodrop (ThermoFisher) and Bioanalyzer (Agilent 2100) instrument. Subsequently, cDNA libraries were constructed for Illumina sequencing on HiSeq 2500 platform. Raw reads were filtered using fastp (v0.20.0) to remove adapters and low-quality bases (quality score < Q20), yielding clean reads. Gene functions were annotated using different databases, including Nr, Nt, Pfam, KOG/COG, Swiss-Prot, KO, and GO.

The clean reads were aligned to the *C. sinensis* vs. Longjing43 reference genome using HISAT2 (v2.2.1), and transcript assembly was performed using StringTie (v2.2.1). Quantification of gene expression levels was evaluated by fragments per kilobase of transcript per million fragments mapped (FPKM). Differentially expressed genes (DEGs) between two conditions were analyzed using DESeq2 software (v1.12.2). The filter criteria for genes with FDR < 0.01 and log_2_FC ≥ 2 were assigned as DEGs. Additionally, the GOseq R package (v2.18.0) was employed to implement GO enrichment analysis of DEGs, and clusterProfiler (v3.10.1) was used to test the statistical enrichment of DEGs in KEGG pathways (significance threshold of *p* < 0.05).

### 2.5 Validation of gene expression by qRT-PCR analysis

The relative mRNA expression levels of six DEGs involved in the alkaloid biosynthesis were validated by qRT-PCR analysis. The sequences of DEGs chosen for qRT-PCR were acquired from the transcriptome data, which the primers were designed using Primer 5 software (**Supplementary Table 1**). Primer efficiency was > 95% for all primer pairs. The reaction system and qRT-PCR protocols employed in this study were identical to those used in the previous investigation. The relative expression level of each target gene was calculated using the method of 2^–ΔΔCt^, with *β*-actin gene of tea serving as an internal standard. The stability of the *β*-actin reference gene was validated by geNorm analysis, showing minimal variation across all samples.

### 2.6 Statistical analysis

All data for qRT-PCR and metabolites were processed by SPSS statistics version 23 and expressed as means ± standard deviation (SD). The statistical significance of the data was evaluated using a one-way analysis of variance (ANOVA), with the least significant difference (LSD) determining the significant differences at a significance level of *p* < 0.05.

## 3 Results

### 3.1 Identification of alkaloid metabolites in tea infected by blister blight

The leaf samples were used to analyze the variations in alkaloid metabolites by UPLC-MS/MS approach and MWDB among different stages of tea following blister blight infection. Principal component analysis (PCA) exhibited distinct patterns of separation between groups and clustering within groups, with 32.26% and 22.22% of the total variance explained by PC1 and PC2, respectively (**Figure 1B**). The quality evaluation of leaf samples through the Pearson Correlation Coefficient (PCC) revealed a significant correlation among duplicated samples within the group for alkaloid metabolites (**Supplementary Figure 1**). In total, 79 metabolites exhibited a clear hierarchical clustering of samples in the heatmap visualization. These substances were divided into eight classes: alkaloids (35), phenolamine (19), plumerane (16), pyridine alkaloids (4), pyrrole alkaloids (2), tropan alkaloid (1), quinoline alkaloid (1), and isoquinoline alkaloid (1) (**Figure 1C**, **Supplementary Table 2**). Furthermore, the OPLS-DA discriminant model based on differential alkaloid metabolites (DAMs) exhibited goodness-of-fit and predictive parameters (R²X = 0.697, R²Y = 1.000, Q² = 0.964), confirming the robustness and validity of the analytical model employed in this study (**Supplementary Figure 2**).

### 3.2 Analysis of alkaloid metabolites across different stages of blister blight infection

**Figure 2:**
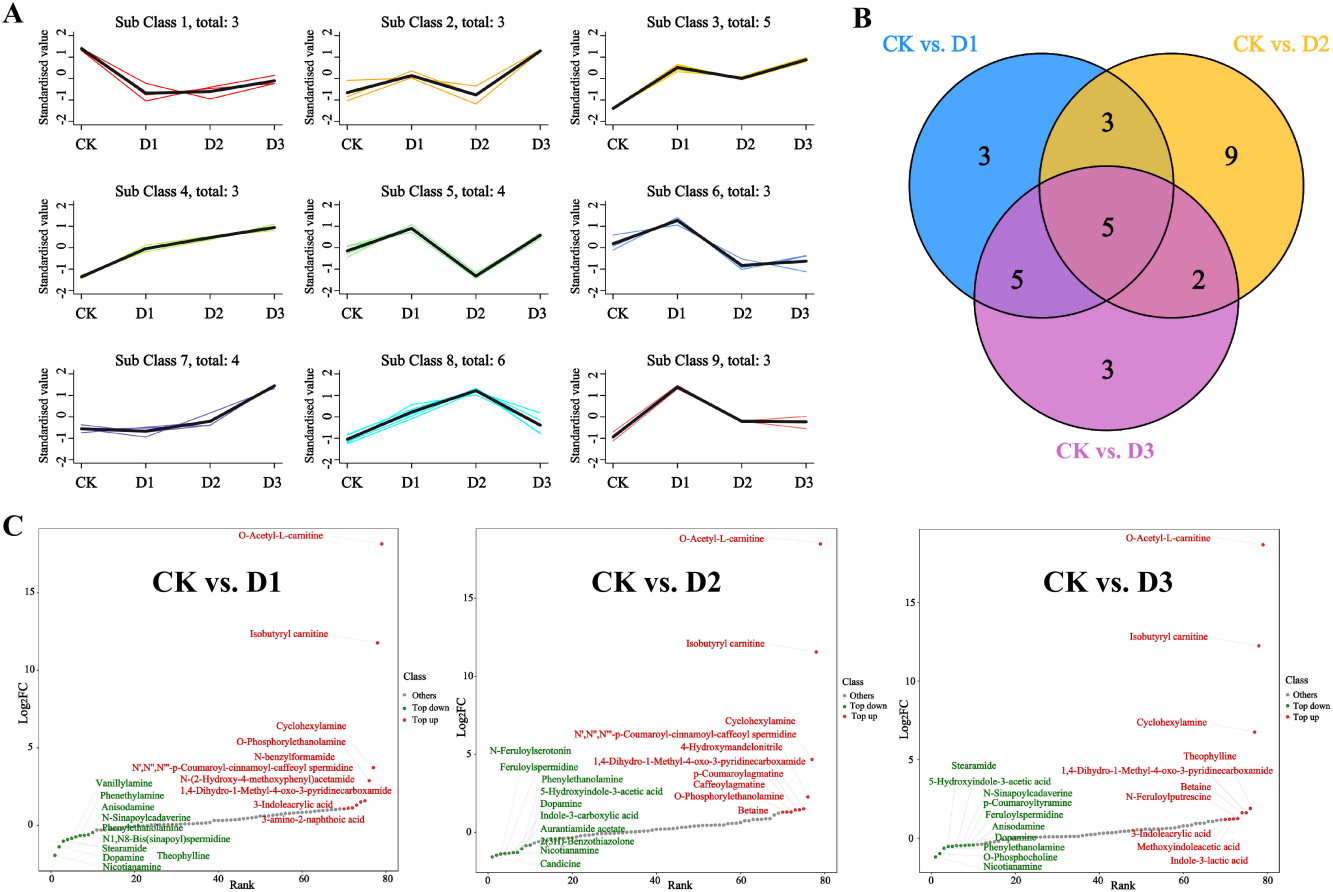
Analysis of differential alkaloid metabolites (DAMs) across different stages of blister blight infection. **(A)** K-means cluster analysis of DAMs. The subclass represents the category numbers of metabolites with the same trend of change. **(B)** Venn diagram of the DAMs. **(C)** The fold change value for top ten up- and down-regulated DAMs in different comparison groups.

To investigate the dynamic changes in DAMs during blister blight progression, the relative abundances of alkaloid metabolites identified were normalized using z-scores in all group comparisons, followed by K-means clustering analysis. From the perspective of metabolite abundance, DAMs exhibited nine distinct patterns of variation across the four stages (**Figure 2A**). Briefly, the abundance of DAMs in Sub Class 1 was relatively higher in the healthy leaves compared to infected leaves, consisting of three alkaloid metabolites involved in phenylethanolamine, dopamine, and nicotianamine. The levels of DAMs demonstrated a similar trend in Sub Class 2, 3, 5, 6 and 9, which were an initial increase followed by a decrease and then a subsequent increase. Among them, the contents of DAMs in the first stage of infection were significantly higher than other stages, mainly including 5,6-dihydroxyindole-5-*O*-*β*-glucoside, *O*-phosphocholine, *N*-benzylformamide, and *O*-phosphorylethanolamine. Additionally, the abundance of DAMs classified in Sub Class 4, 7 and 8 exhibited the increasing trends in variation within the infected leaves; particularly, the highest growth rates were observed for *N’*, *N’’*, *N’’’*-p-coumaroyl-cinnamoyl-caffeoyl spermidine, belonging to phenylamine, and theophylline, belonging to alkaloids, with increases of 3.82 and 2.72 times, respectively.

The Venn diagram showed that a total of 30 DAMs were shared in the three comparison groups (**Figure 2B**). Among them, 16 (13 up-regulated and 3 down-regulated), 19 (12 up-regulated and 7 down-regulated), and 15 (14 up-regulated and 1 down-regulated) DAMs were identified in CK vs. D1, CK vs. D2, and CK vs. D3, respectively (**Supplementary Table 3**). Additionally, five common DAMs involved in cyclohexylamine, *O*-acetyl-*L*-carnitine, isobutyryl carnitine, nicotianamine, and 1,4-dihydro-1-methyl-4-oxo-3-pyridinecarboxamide, most of which belong to the category of alkaloids, were found in the comparisons between CK vs. D1, CK vs. D2, and CK vs. D3 (**Figure 2C**, **Supplementary Table 3**). The FC value for DAMs exhibiting the most significant difference among the top ten up- and down-regulated substances were associated with alkaloids and phenolamines. Among these, *O*-acetyl-*L*-carnitine displayed the highest up-regulation in terms of relative abundance (log_2_FC > 18.67), followed by isobutyryl carnitine (log_2_FC > 12.24) in CK vs. D3. Whereas the relative abundance of nicotianamine exhibited the highest down-regulation (log_2_FC < -1.93) in CK vs. D1. KEGG pathway enrichment revealed that alkaloid metabolites were enriched in 16 metabolic pathways among three comparison groups of CK vs. D1, CK vs. D2, and CK vs. D3, such as nicotinate and nicotinamide metabolism, sphingolipid metabolism, tyrosine metabolism, glycolysophosphatidylinositol (GPI) biosynthesis, isoquinoline alkaloid biosynthesis, cysteine and methionine metabolism (**Supplementary Figure 3**).

### 3.3 Illumina sequencing and assembly of functional annotation in tea transcriptome

**Figure 3:**
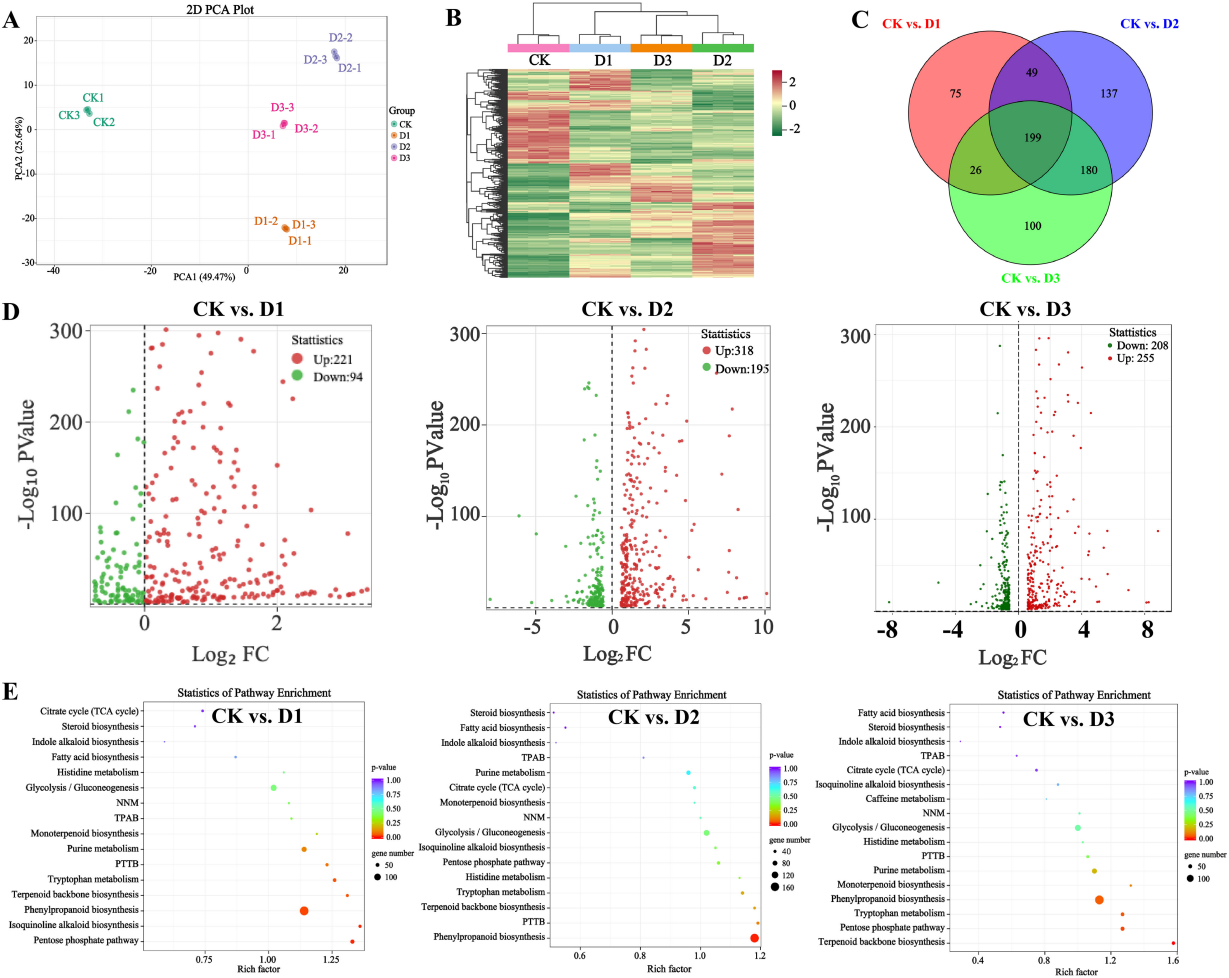
Analysis of differential expressed genes (DEGs) and enriched metabolic pathways across different stages of blister blight infection. **(A)** Principal component analysis (PCA) of different stages of infection regarding their gene expression. **(B)** Heatmap of the DEGs following blister blight disease. **(C)** Venn diagram of DEGs. **(D)** Volcano plot of the DEGs in three comparisons. **(E)** KEGG pathway enrichment of DEGs. TPAB, tropane, piperidine and pyridine alkaloid biosynthesis; NNM, nicotinate and nicotinamide metabolism; PTTB, phenylalanine, tyrosine and tryptophan biosynthesis.

PCA showed that intra-group biological replicates exhibited close clustering, whereas inter-group samples displayed significant segregation, confirming the robustness and reproducibility of the experimental data (**Figure 3A**). Briefly, healthy leaf samples were clearly separated from infected samples along PC1, which accounted for 49.47% of the total variance, and from D1 and D2 along PC2, which explained an additional 25.64%. These results suggest that blister blight infection induce substantial metabolic reprogramming in tea leaves. In total, 18,650 differentially expressed genes (DEGs) were identified in this study. These DEGs exhibited significant differences in expression between groups D1, D2, and D3 compared to the CK group (**Figure 3B**), which aligned with the observed accumulation of DAMs. Using thresholds of |log_2_FC| ≥ 1 and FDR < 0.05, a total of 846 DEGs were identified across the three comparison groups. Specifically, 315 (221 up, 94 down), 513 (318 up, 195 down), and 463 (255 up, 208 down) DEGs were detected in D1, D2, and D3, respectively, relative to CK (**Figure 3D**). Additionally, 199 common DEGs were observed in CK vs. D1, CK vs. D2, and CK vs. D3 (**Figure 3C**).

The Go functional annotation revealed that 335, 542, and 479 DEGs identified from CK vs. D1, CK vs. D2, and CK vs. D3 were categorized into 33, 35, and 32 terms, respectively (**Supplementary Table 4**). In the category of biological process, the most commonly annotated terms for DEGs were “metabolic process” and “single-organism process”, with 335 and 278 DEGs, respectively, in the comparison group of CK vs. D2 (**Supplementary Figure 4A**). In the category of molecular function, the most abundant terms for DEGs annotations were “catalytic activity” and “binding”, with a maximum of 480 and 240 DEGs, respectively, in D2 group compared with CK group (**Supplementary Figure 4B**). In the category of cellular component, the “cell part” and “cell” were the terms of the highest number for DEGs annotations, with 83 DEGs each between CK and D2 groups (**Supplementary Figure 4C**). KEGG enrichment and functional annotation of DEGs showed that 348, 564, and 504 unigenes from the comparisons CK vs. D1, CK vs. D2, and CK vs. D3 were annotated into 69, 72, and 75 pathways, respectively (**Supplementary Table 5**). Among these, the majority of DEGs were significantly enriched in the metabolic pathways of phenylpropanoid biosynthesis, pentose and glucuronate interconversions, isoquinoline alkaloid biosynthesis, terpenoid backbone biosynthesis, and tyrosine metabolism (**Figure 3E**). The enrichment of isoquinoline alkaloid biosynthesis is particularly noteworthy, as this pathway is central to the production of defense-related alkaloids in many plant species. In addition, three metabolic pathways, including caffeine metabolism, oxidative phosphorylation, and ribosome biogenesis in eukaryotes, were specifically enriched in CK vs. D3 (**Supplementary Table 5**). Beyond enrichment, the co-occurrence of isoquinoline alkaloid and phenylpropanoid pathways indicated a multilayered defense: alkaloids as chemical barriers and phenylpropanoids for physical/oxidative reinforcement. The late-stage enrichment of ribosome/oxidative phosphorylation suggested a shift from active metabolite production to cellular maintenance, revealing a temporal resource-reallocation.

### 3.4 Integrated analysis of the transcriptome and metabolome of tea leaves responsive to blister blight infection

**Figure 4:**
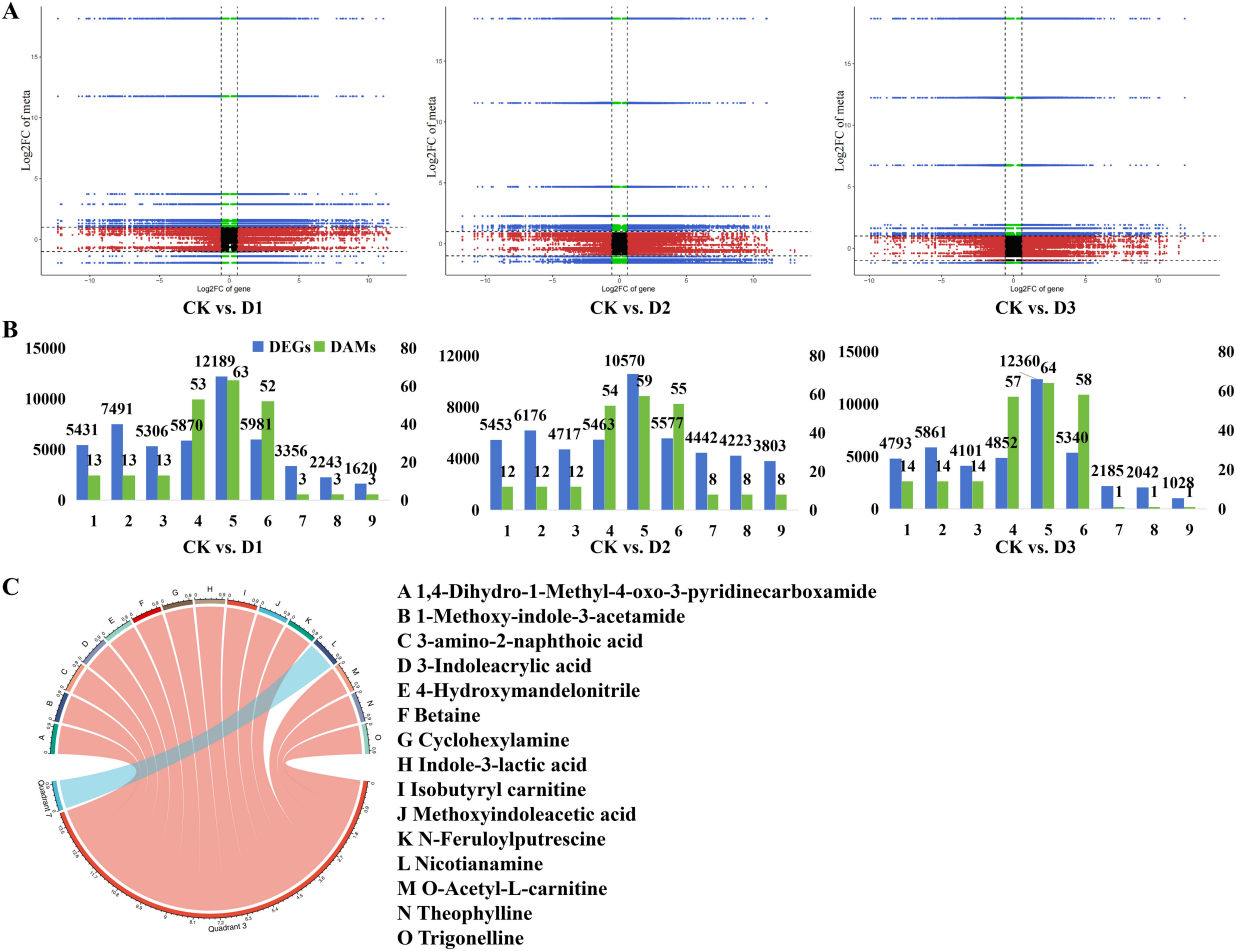
Integrated analysis of DAMs and DEGs of tea leaves responsive to blister blight infection. **(A)** Nine-quadrant map of genes and alkaloid metabolites. **(B)** The number of DAMs and DEGs in each quadrant. **(C)** DAMs in quadrant 3 and quadrant 7.

To illustrate the correlations between DEGs and DAMs, nine quadrant diagrams were constructed based on Pearson correlation coefficients greater than 0.8 (**Figure 4A**). In the nine-quadrant correlation plots, genes and metabolites with no differential abundance were located in quadrant 5. Genes and metabolites situated in both quadrants 3 and 7 displayed a coincident pattern of differential expression, suggesting a potential positive regulation of metabolites expressions by genes; whereas those positioned in quadrants 1 and 9 demonstrated negative correlations in their regulation. Metabolites located in quadrants 2 and 8 exhibited an up-regulated or down-regulated trend, while genes remained unchanged. Conversely, genes and metabolites located in quadrants 4 and 6 showed the opposite phenomenon, that is, genes were up-regulated or down-regulated, but metabolites remained unchanged.

Among all comparisons, CK vs. D3 yielded the highest number of features in quadrant 6, with 12,360 genes and 64 metabolites (**Figure 4B**). Notably, DAMs located in quadrants 3 and 7 were likely regulated by their respective DEGs. In CK vs. D1, 13 DAMs and 5,306 DEGs were found to be up-regulated in quadrant 3, and 3 DAMs and 3,356 DEGs were down-regulated in quadrant 7. In CK vs. D2, quadrant 3 comprised 12 DAMs and 4,717 DEGs (both up-regulated), whereas quadrant 7 included 8 DAMs and 4,442 DEGs (both down-regulated). In CK vs. D3, quadrant 3 contained 14 DAMs and 4,101 DEGs (up-regulated), and quadrant 7 contained 1 DAM and 2,185 DEGs (down-regulated) (**Figure 4B**).

To further dissect the gene–metabolite interplay at the late stage of blister blight infection, we examined quadrants 3 and 7 in the CK vs. D3 comparison. Among the features in quadrant 7, the alkaloid metabolite nicotianamine was significantly down-regulated by DEGs, while 14 DAMs in quadrant 3 including cyclohexylamine, betaine, 4-hydroxymandelonitrile, 3-indoleacrylic acid, 1,4-dihydro-1-methyl-4-oxo-3-pyridinecarboxamide, 1-methoxy-indole-3-acetamide, and theophylline exhibited positive regulation with DEGs (**Figure 4C**).

### 3.5 Analysis of DEGs and DAMs involved in alkaloid metabolic network

**Figure 5:**
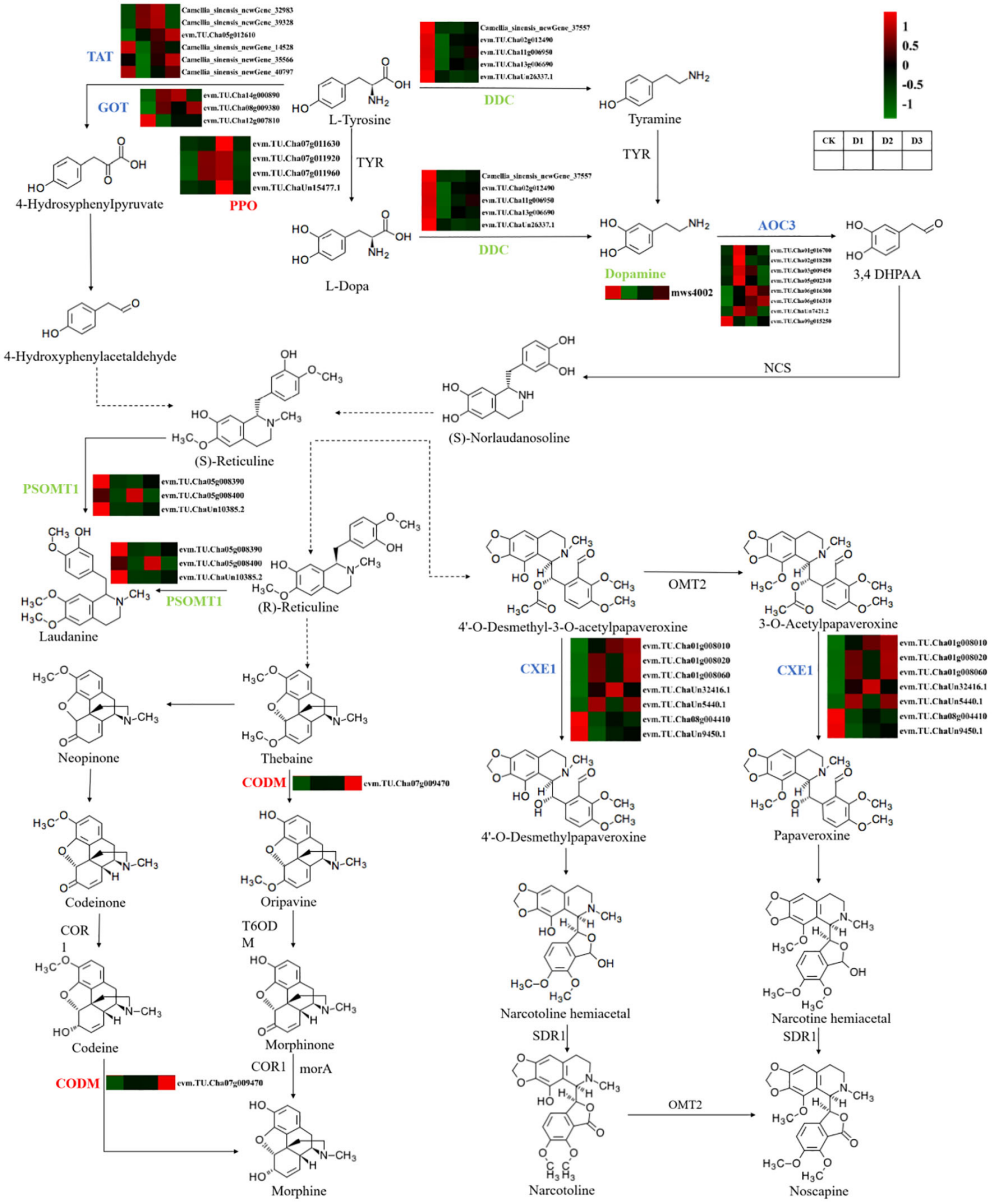
Analysis of DEGs and DAMs involved in isoquinoline alkaloid biosynthesis. TAT, tyrosine aminotransferase; GOT, aspartate aminotransferase; PPO, polyphenol oxidase, DDC, aromatic-*L*-amino-acid; AOC3, primary-amine oxidase; PSOMT1, (*R,S*)-reticuline 7-*O*-methyltransferase; CODM, codeine 3-*O*-demethylase; CXE1, 3-*O*-acetylpapaveroxine carboxylesterase.

Our integrated transcriptomic and metabolomic analysis confirmed that the isoquinoline alkaloid biosynthesis significantly contributes to the immune response of tea plants against blister blight. We therefore investigated the relationships between putative enzyme-encoding genes, structural genes involved in critical intermediate compounds, and their interactions with key metabolites within this pathway. In total, 37 genes and one metabolite were significantly annotated to the isoquinoline alkaloid biosynthesis pathway (**Figure 5**, **Supplementary Table 6**). Among these, tyrosine aminotransferase (*TAT*), aspartate aminotransferase (*GOT*), polyphenol oxidase (*PPO*), aromatic-*L*-amino-acid decarboxylase (*DDC*), and primary-amine oxidase (*AOC3*) were identified as crucial genes in the upstream synthesis of isoquinoline alkaloids. From *L*-tyrosine onward, 17 out of the 26 genes encoding these enzymes, including four *TATs* (*Camellia_sinensis_newGene_32983*, *Camellia_sinensis_newGene_39328*, *evm.TU.Cha05g012610*, and *Camellia_sinensis_newGene_35566*), two *GOTs* (*evm.TU.Cha14g000890* and *evm.TU.Cha08g009380*), four *PPOs* (*evm.TU.Cha07g011630*, *evm.TU.Cha07g011920*, *evm.TU.Cha07g011960*, and *evm.TU.ChaUn15477.1*), and seven *AOC3* (*evm.TU.Cha01g016700*, *evm.TU.Cha02g018280*, *evm.TU.Cha03g009450*, *evm.TU.Cha05g002340*, *evm.TU.Cha06g016300*, *evm.TU.Cha06g016310*, and *evm.TU.ChaUn7421.2*) were significantly upregulated across the entire stage of infection (**Figure 5**). Among these 17 upregulated DEGs, the four genes with the highest log_2_FC values were two *TATs* (*Camellia_sinensis_newGene_32983* and *Camellia_sinensis_newGene_39328*) and two *PPOs* (*evm.TU.Cha07g011630* and *evm.TU.ChaUn15477.1*), with values of 8.78, 7.54, 7.62, and 6.83, respectively, in the CK vs. D2 (**Supplementary Table 6**). In contrast, nine genes encoding one *GOT* (*evm.TU.Cha12g007810*), two *TATs* (*Camellia_sinensis_newGene_14528* and *Camellia_sinensis_newGene_40797*), five *DDCs* (*evm.TU.Cha02g012490*, *evm.TU.Cha11g006950*, *evm.TU.Cha13g006690*, *Camellia_sinensis_newGene_37557*, and *evm.TU.ChaUn26337.1*), and one *AOC3* (*evm.TU.Cha09g015250*) were downregulated in various comparisons, with the two *TAT* genes (*Camellia_sinensis_newGene_14528* and *Camellia_sinensis_newGene_40797*) showing the most pronounced decreases (log_2_FC = –3.84 and –1.96 in CK vs. D1; **Figure 5**; **Supplementary Table 6**). Additionally, the expression of (*R,S*)-reticuline 7-*O*-methyltransferase (*PSOMT1*), codeine 3-*O*-demethylase (CODM), and 3-*O*-acetylpapaveroxine carboxylesterase (*CXE1*) were linked to the downstream of isoquinoline alkaloid biosynthesis pathway. Six of the 11 genes encoding *CODM* (*evm.TU.Cha07g009470*) and *CXE1* (*evm.TU.Cha01g008010*, *evm.TU.Cha01g008020*, *evm.TU.Cha01g008060*, *evm.TU.ChaUn32416.1*, and *evm.TU.ChaUn5440.1*) were significantly upregulated throughout infection, with the *CODM* gene (*evm.TU.Cha07g009470*) exhibiting a particularly high log_2_FC of 5.65 in CK vs. D2. Conversely, almost three *PSOMT1* genes (*evm.TU.Cha05g008390*, *evm.TU.Cha05g008400*, and *evm.TU.ChaUn10385.2*) and two *CXE1* genes (*evm.TU.Cha08g004410* and *evm.TU.ChaUn9450.1*) were markedly downregulated in the downstream pathway. In terms of metabolite abundance, the relative level of dopamine was significantly reduced across all infection stages, with the most prominent decrease observed in CK vs. D1.

### 3.6 Transcription factors analysis involved in alkaloid metabolites

**Figure 6:**
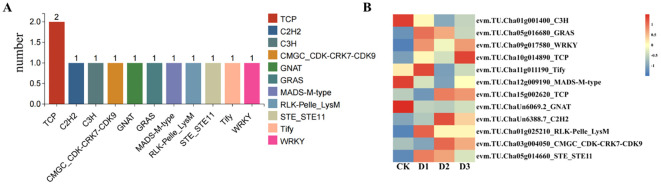
Transcript factors (TFs) of DEGs involved in the synthesis of alkaloids. **(A)** The number of TFs. **(B)** The expression of TFs.

Transcription factors (TFs) regulate various physiological processes in plants, including germination, metabolism, and the response to biotic and abiotic stresses (Huang et al., 2023). In this study, a total of 12 differentially expressed TFs, belonging to 11 TF families, were annotated to be associated with the alkaloid biosynthesis pathway (**Figure 6A**). Among these, eight genes encoding *GRAS* (*evm.TU.Cha05g016680*), *WRKY* (*evm.TU.Cha09g017580*), *TCP* (*evm.TU.Cha10g014890* and *evm.TU.Cha15g002620*), *C2H2* (*evm.TU.ChaUn6388.7*), *RLK-Pelle_LysM* (*evm.TU.Cha01g025210*), *CMGC_CDK-CRK7-CDK9* (*evm.TU.Cha03g004050*), and *STE_STE11* (*evm.TU.Cha05g014660*) were upregulated, whereas four genes encoding *C3H* (*evm.TU.Cha01g001400*), *Tify* (*evm.TU.Cha11g011190*), *MADS-M-type* (*evm.TU.Cha12g009190*), and *GNAT* (*evm.TU.ChaUn6069.2*) were downregulated (**Figure 6B**). These families are intimately involved in the synthesis of carrier alkaloids. Notably, *WRKY* TFs constitute a large family of regulatory proteins recognized for their significant role in modulating plant immunity (Pandey and Somssich, 2009). Hence, the substantial accumulation of WRKY transcripts in tea leaves following blister blight infection may contribute to the resistance of tea against this pathogen.

### 3.7 qRT-PCR validation of DEG expression

**Figure 7:**
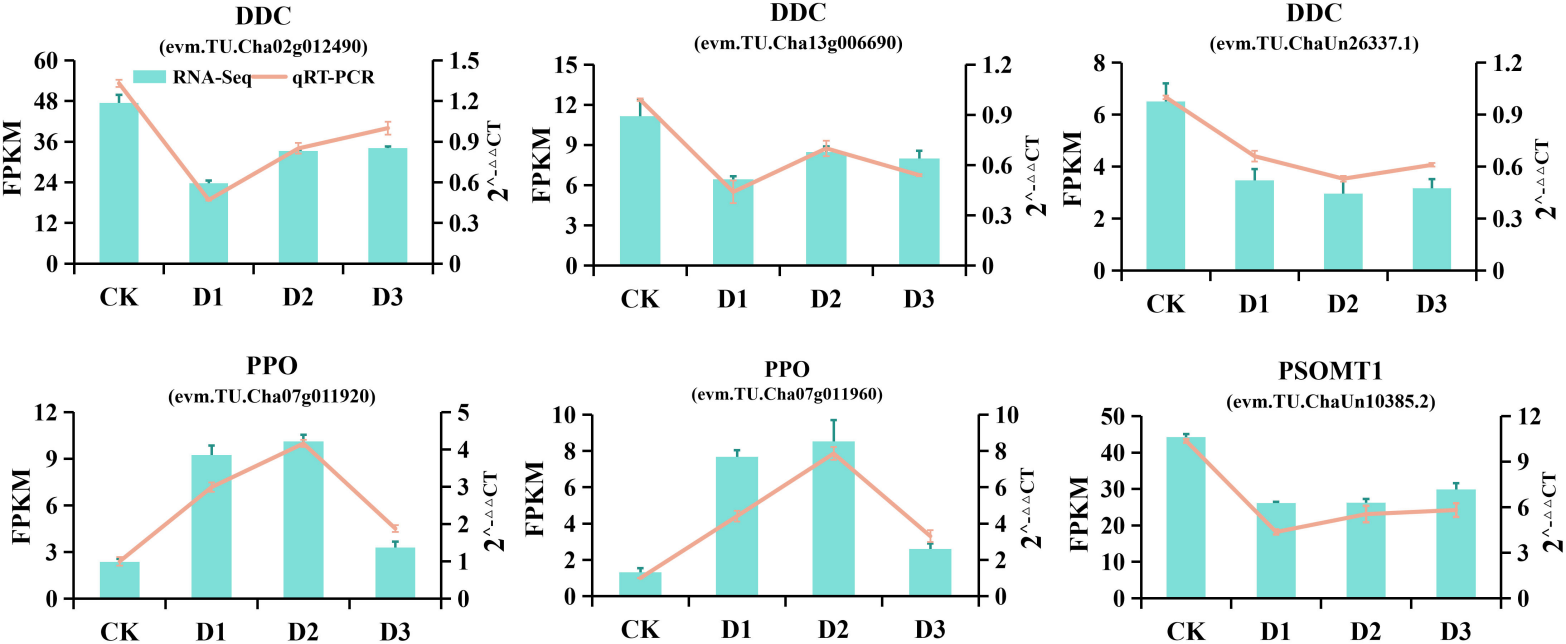
qRT-PCR validation of DEGs associated with isoflavonoid biosynthesis at different stages of blister blight infection. A total of six DEGs involve in genes encoding *DDC*, *PPO*, and *PSOMT1*.

qRT-PCR analysis was performed to validate the expression profiles of six genes encoding crucial enzymes involved in alkaloid biosynthesis in healthy and *E. vexans*-infected leaves. The results demonstrated that the expression levels of six DEGs including four downregulated genes encoding *DDC* (*evm.TU.Cha02g012490*, *evm.TU.Cha13g006690*, and *evm.TU.ChaUn26337.1*) and *PSOMT1* (*evm.TU.ChaUn10385.2*), and two upregulated genes encoding *PPO* (*evm.TU.Cha07g011920* and *evm.TU.Cha07g011960*) were consistent with their corresponding FPKM values obtained from RNA-Seq analysis (**Figure 7**). The strong concordance between the qRT-PCR and RNA-Seq datasets confirms the reliability and robustness of the transcriptomic sequencing data.

## 4 Discussion

Plants are frequently subjected to various stressors, resulting in significant agricultural yield losses. Thus, a comprehensive understanding of the immune mechanisms underlying tea plant resistance against blister blight is essential for implementing effective resistance breeding programs aimed at enhancing multiple stress tolerance in tea cultivars.

Within plant immune responses, secondary metabolites serve as a biochemical barrier against pathogen invasion (Luna et al., 2011). Phytoalexins are important secondary metabolites involved in plant defense against pathogen infection (Dixon, 2001), and they accumulate rapidly during infection to inhibit pathogen growth and reproduction, thereby playing a crucial role in disease resistance (Jeffrey, 1999). To date, over 200 phytoalexins have been identified, with the most prominent classes including flavonoids, terpenoids, indoles, coumarins, saponins, and alkaloids. Among them, alkaloids are predominant secondary metabolites that bolster plant responses to both biotic and abiotic stresses across numerous species (Frick et al., 2018). However, research on the role of alkaloids in tea plant defense against fungal pathogens remains relatively limited. In the present study, a total of 79 alkaloid metabolites were identified across different stages of blister blight infection, predominantly belonging to the categories of alkaloids, phenolamines, plumeranes, pyridine alkaloids, pyrrole alkaloids, tropane alkaloids, quinoline alkaloids, and isoquinoline alkaloids. Notably, the majority of these alkaloid metabolites were upregulated in tea leaves following blister blight infection, consistent with previous reports demonstrating that significant accumulation of alkaloid metabolites was associated with disease resistance in plant–pathogen interactions (Zhou et al., 2023). Similar accumulation patterns have also been observed in cotton infected with *Verticillium wilt* (Zhang et al., 2023) and in *Camellia oleifera* infected with *Colletotrichum gloeosporioides* (Yang et al., 2022). Notably, five common DAMs, including 1,4-dihydro-1-methyl-4-oxo-3-pyridinecarboxamide, cyclohexylamine, isobutyryl carnitine, *O*-acetyl-*L*-carnitine, and nicotianamine, related to alkaloids were shared among the three comparisons (CK vs. D1, D2, and D3), indicating that these five metabolites may be continuously involved in the tea plant’s defense response against blister blight. These conserved metabolites represent promising biomarker candidates for blight resistance in breeding programs.

We identified 846 significant genes across the different stages of infection, with a greater number of upregulated than downregulated DEGs observed in three comparison groups. This finding is consistent with the result of Rui et al. (2022), suggesting that pathogen infection triggers the transcriptional activation of most genes. Additionally, we observed that majority of DEGs were commonly enriched in the isoquinoline alkaloid biosynthesis pathway, suggesting their critical involvement in the accumulation of isoquinoline alkaloids. The biosynthesis of isoquinoline alkaloids initiates with the conversion of *L*-tyrosine into dopamine and 4-hydroxyphenylacetaldehyde, which subsequently undergo intramolecular coupling, reduction, methylation, hydroxylation, and other reactions to generate a diverse array of structurally complex compounds (Yang et al., 2017). Isoquinoline alkaloids, as the antifungal compound, extracted from *Corydalis ternata* displayed antifungal activity against wheat leaf rust and pepper anthracnose (Han et al., 2018). Additionally, the biosynthesis responsible for numerous isoquinoline alkaloids have been identified in various plants (Li et al., 2024), for instance, reported that the biosynthesis of isoquinoline alkaloids may enhance tolerance to cadmium by serving as a crucial pathway in cadmium response. Whereas the immune mechanism of tea plant in response to pathogen infection remains unclear, mainly due to limited genetic information. Consequently, we analyzed the expression patterns of key genes and metabolites involved in isoquinoline alkaloid biosynthesis. The initial enzymes including hydroxyphenylacetaldehyde synthase (*TYRDC*), *L*-amino-acid oxidase (*LAAO*), *TYR*, *TAT*, *GOT*, *PPO*, and *DDC* play a crucial role in mediating the synthesis of upstream isoquinoline alkaloid derivatives (Peng et al., 2020). Among these, *TAT* and *GOT* are potentially involved in catalyzing the conversion of 4-hydroxyphenylpyruvate, which subsequently leads to the formation of coclaurine and ultimately yields reticuline and scoulerine. Additionally, *PPO* and *DDC* facilitate the conversion of *L*-tyrosine to dopamine; notably, *DDC* catalyzes the initial step in the production of bioactive plant compounds, a function that has been extensively studied in plants (Torrens-Spence et al., 2018). In the present study, a total of 37 structural genes and enzymes involved in isoquinoline alkaloid biosynthesis (e.g., *TAT*, *GOT*, *AOC3*, *CXE1*, and *CODM*) exhibited significant differential expression during blister blight infection, suggesting that these changes may enhance alkaloid biosynthesis and accumulation, thereby contributing to the tea plant’s defense response against the pathogen. We also acknowledge that functional annotations in this study are based on sequence homology and KEGG orthology assignments, which do not guarantee equivalent biochemical functions in *C. sinensis*. Enzymes such as *PSOMT1* and *CODM*, while well-characterized in opium poppy and other isoquinoline alkaloid-producing plants (Hagel and Facchini, 2010), may have different substrate specificities or catalytic activities in tea. Future studies involving heterologous expression, enzyme activity assays, or CRISPR/Cas9-mediated gene knockout are required to confirm the precise functions of these candidate genes.

Importantly, dopamine, a critical precursor in isoquinoline alkaloid biosynthesis (Kulma and Szopa, 2006), significantly downregulated at all infection stages despite the upregulation of synthetic genes (e.g., *TAT*, *PPO*). We therefore propose a mechanistic model wherein this inverse correlation is driven by enhanced metabolic flux. Specifically, pathogen-induced upregulation of upstream genes elevates dopamine biosynthesis; however, this pool is rapidly consumed and channeled into downstream isoquinoline branches by the action of norcoclaurine synthase (NCS) (Samanani and Facchini, 2002; Both et al., 2006). Hence, the net decrease in dopamine abundance is attributable to a downstream ‘pull’ exerted by defense-related alkaloid biosynthesis, which surpasses the rate of *de novo* synthesis. Although our data strongly suggest this active channeling, we cannot exclude the possibility of additional regulatory mechanisms such as feedback inhibition of *TAT* or *DDC* by accumulating end-products, or post-translational modifications that could fine-tune enzyme activity (Kulma and Szopa, 2006). Ultimately, this dynamic interplay between transcriptional activation and metabolic turnover highlights a key regulatory checkpoint in plant defense, with dopamine downregulation serving as a metabolic signature of induced resistance.

Transcription factors (TFs) play crucial roles in regulating defense-associated genes in plant immune responses (Tweneboah and Oh, 2017). In this study, a total of 12 TFs involved in alkaloids biosynthesis were identified during infection, including the upregulation of *WRKY*, *GRAS*, and *C2H2*, alongside the downregulation of *C3H* and *Tify*. Previous studies have established these families as regulators of stress and metabolism (Jayaswall et al., 2016; Jiang et al., 2017; Deng et al., 2023; Lu et al., 2023). However, our correlative analysis provides a distinct context-specific insight: the inverse expression trends within these families are uniquely coupled with the isoquinoline-specific metabolic flux shift observed here. Notably, while *WRKY* and *GRAS* have previously been associated with alkaloid regulation in other species (Day et al., 2003; Kato et al., 2007), their co-induction with structural genes (e.g., *TAT* and *PPO*) in our study suggested a synergistic regulatory module specifically configured to blister blight defense. Conversely, the downregulation of *C3H* and *Tify* in our infected leaves implied their potential role as negative regulators in this particular pathosystem. Based on these correlations, we propose a working model in which upregulated TFs (*WRKY*, *GRAS*) may positively regulate structural genes (*TAT*, *PPO*, *AOC3*), while downregulated TFs (*C3H*, *MADS-M-type*) may exert negative regulatory effects. This establishes a regulatory axis of ‘TF activation, structural gene upregulation, metabolic precursor modulation’, which may promote the accumulation of downstream isoquinoline alkaloids to combat pathogen invasion.

However, we acknowledge several limitations in the current study. First, the metabolomic platform employed (UPLC-MS/MS with the MWDB database) is widely targeted and may not comprehensively capture all isoquinoline alkaloid derivatives, particularly low-abundance end products such as reticuline, papaverine, or berberine. The detection of only one pathway-associated metabolite (dopamine) limits our ability to definitively confirm complete pathway activation. Second, while the transcriptional upregulation of structural genes and the downregulation of the precursor dopamine are consistent with increased metabolic flux, direct biochemical evidence of enhanced isoquinoline alkaloid production, such as targeted LC-MS/MS with authentic standards or isotope tracing studies, is needed to validate this inference. Third, the regulatory relationships inferred between TFs and structural genes are correlational and require experimental validation through methods such as ChIP-seq, yeast one-hybrid assays, or transient overexpression. Furthermore, the assignment of gene functions based on sequence similarity, particularly for downstream enzymes such as *PSOMT1*, *CODM*, and *CXE1*, awaits experimental confirmation through heterologous expression or *in vitro* activity assays. Future research integrating targeted metabolomics and functional gene validation will be essential to fully elucidate the precise role of isoquinoline alkaloids in tea immunity against blister blight.

Overall, this study represents the first comprehensive multi-omics investigation specifically targeting alkaloid metabolism in tea during blister blight infection. The identification of 30 shared DAMs and 199 shared DEGs provides a core set of candidate biomarkers for marker-assisted selection in tea breeding. Key structural genes (*TAT*, *PPO*, *AOC3*, *CODM*) and TFs (*WRKY*, *GRAS*, *C2H2*) identified here can serve as potential targets for genetic improvement of blight resistance. Furthermore, the metabolites identified (e.g., theophylline, nicotianamine) could be explored as natural resistance inducers or as quality markers for disease-resistant cultivars.

## 5 Conclusion

This study presents the first comprehensive analysis of genes and metabolites associated with alkaloid biosynthesis in tea following the invasion of blister blight disease. We identified diverse alkaloid metabolites at multiple infection stages and confirmed the expression of candidate genes involved in alkaloid biosynthesis. These results revealed that the isoquinoline alkaloid pathway was markedly reprogrammed during blister blight infection, suggesting its functional role in tea immunity. The discovery was corroborated by the expression levels of crucial candidate genes involved in alkaloids biosynthesis, as confirmed through qRT-PCR analysis. Our current findings offer valuable insight into the response mechanism of tea against blister blight, potentially facilitating the development of resistant cultivars to manage this disease in tea plant. The precise mechanism through which alkaloids regulate the immunity response of tea to blister blight infection remains incompletely understood and requires further investigation in future research, particularly through targeted metabolomics and functional gene validation studies.

## Data Availability

The original contributions presented in the study are included in the article/supplementary material. Further inquiries can be directed to the corresponding authors.
