# Supplementary material for: Integrated transcriptomic and metabolomic analyses reveal the regulatory network of alkaloid metabolism in blister blight-infected tea plants
Source: Front Plant Sci. 2026 Aug 5;17:1885792. doi: 10.3389/fpls.2026.1885792 (PMC13486230; doi:10.3389/fpls.2026.1885792)
Supplement: Supplementary file 1 [file DataSheet1.zip › Supplementary files/Supplementary figures.docx]

**Figure S1.** The Pearson correlation coefficient of the metabolite expression profile

**Figure S2.** Validation OPLS-DA model of DAMs in the three comparison groups. R^2^X and R^2^Y quantify the proportions of explained variance in the X and Y matrices by the model, respectively. Q^2^ assesses the model's predictive capability. A higher value approaching 1 in these three indicators indicates a more stable and reliable model. A model is considered effective when Q^2^ > 0.5, and superior when Q^2^ > 0.9.

**Figure S3.** KEGG pathway enrichment analysis of DAMs in (**A**) CK vs. D1, (**B**) CK vs. D2, (**C**) CK vs. D3. The x-axis represents the enrichment degree, and the y-axis represents the enriched pathways; the color of each data point denotes the corresponding *P*-value.

**Figure S4.** Go functional annotation of DEGs in (**A**) CK vs. D1, (**B**) CK vs. D2, (**C**) CK vs. D3. BP, biological process categories; CC, component categories category; MF, molecular function category.
